# Application of Texture Analysis Based on Sagittal Fat-Suppression and Oblique Axial T2-Weighted Magnetic Resonance Imaging to Identify Lymph Node Invasion Status of Rectal Cancer

**DOI:** 10.3389/fonc.2020.01364

**Published:** 2020-08-07

**Authors:** Lirong Song, Jiandong Yin

**Affiliations:** Department of Radiology, Shengjing Hospital of China Medical University, Shenyang, China

**Keywords:** rectal cancer, texture analysis, lymph node invasion, magnetic resonance imaging, radiomics

## Abstract

**Objective:** To investigate the value of texture features derived from T2-weighted magnetic resonance imaging (T2WI) for predicting preoperative lymph node invasion (N stage) in rectal cancer.

**Materials and Methods:** One hundred and eighty-two patients with histopathologically confirmed rectal cancer and preoperative magnetic resonance imaging were retrospectively analyzed, who were divided into high (N1-2) and low N stage (N0). Texture features were calculated from histogram, gray-level co-occurrence matrix, and gray-level run-length matrix from sagittal fat-suppression and oblique axial T2WI. Independent sample *t*-test or Mann-Whitney *U*-test were used for statistical analysis. Multivariate logistic regression analysis was conducted to build the predictive models. Predictive performance was evaluated by receiver operating characteristic (ROC) analysis.

**Results:** Energy (ENE), entropy (ENT), information correlation (INC), long-run emphasis (LRE), and short-run low gray-level emphasis (SRLGLE) extracted from sagittal fat-suppression T2WI, and ENE, ENT, INC, low gray-level run emphasis (LGLRE), and SRLGLE from oblique axial T2WI were significantly different between stage N0 and stage N1-2 tumors. The multivariate analysis for features from sagittal fat-suppression T2WI showed that higher SRLGLE and lower ENE were independent predictors of lymph node invasion. The model reached an area under ROC curve (AUC) of 0.759. The analysis for features from oblique axial T2WI showed that higher INC and SRLGLE were independent predictors. The model achieved an AUC of 0.747. The analysis for all extracted features showed that lower ENE from sagittal fat-suppression T2WI and higher INC and SRLGLE from oblique axial T2WI were independent predictors. The model showed an AUC of 0.772.

**Conclusions:** Texture features derived from T2WI could provide valuable information for identifying the status of lymph node invasion in rectal cancer.

## Introduction

Colorectal cancer is the fifth leading cause of cancer-related mortality in China, and rectal cancer accounts for ~30–35% of colorectal cancer cases (1, 2). Treatment methods for rectal cancer include surgery, chemoradiation, and targeted therapy. The status of lymph node invasion (as N stage) is an important prognostic factor for local recurrence and overall survival (3). The National Comprehensive Cancer Network rectal cancer guidelines recommend neoadjuvant chemoradiotherapy (NAT) for patients with lymph node invasion before surgery (4). Therefore, preoperative identification of lymph node invasion status in patients with rectal cancer is crucial for tailoring treatment strategies.

High-resolution magnetic resonance imaging (MRI) is strongly recommended by the American Society of Colon and Rectal Surgeons before treatment because of its ability to non-invasively evaluate the microcirculation of tumors (4, 5). However, the accuracy of detecting lymph node invasion status with morphological criteria such as short-axis diameter, shape, border smoothness and signal heterogeneity is unsatisfactory (6, 7). MRI images are data more than pictures, and they can provide more than morphological information (8). More advanced and reliable techniques may be important for identifying the status of lymph node invasion with rectal MRI images.

Texture analysis, which has emerged as a “radiomics” approach for interpretation of medical imaging, is widely used to characterize the spatial distribution of gray-level intensity in images. Texture analysis captures image patterns that are usually unrecognizable or unresolved by the human eye (9, 10). It extracts high-throughput information to characterize image heterogeneity in specific target regions and is an indicator of tumor heterogeneity (11). MRI has multiparametric imaging ability and can provide more valuable data for radiomics than monomodality imaging methods such as computed tomography (CT) by high-throughput extraction of quantitative image features (12). Texture features derived from MRI images have been proven to be helpful in assessing tumors (13–15). Previous studies indicated that texture features show potential for distinguishing benign from malignant lesions for breast cancer (16). In addition, texture features are useful for evaluating tumor heterogeneity in glioma and cervical cancers (17, 18). For rectal cancer, the application of texture analysis has mainly focused on treatment response to NAT, and earlier findings revealed that texture features were useful for assessment of pathological complete response after NAT (9, 19–21).

First-order texture features, also known as histogram features, quantitatively describe the distribution of pixel signal intensity within a target region. Second-order texture features including gray-level co-occurrence matrix features and gray-level run-length matrix features, consider the distribution of pixel pairs within the target region (22). A previous study (23) showed that histogram features of rectal cancer on MRI were correlated with lymph node status. However, second-order texture features were not used in the study analysis. Moreover, only routine axial non-fat-suppression T2-weighted MRI (T2WI) images were applied for lesion extraction and texture analysis. Therefore, the purpose of this study was to investigate if texture features including histogram and second-order texture features derived from preoperative sagittal fat-suppression and oblique axial T2WI are valuable for predicting the status of lymph node invasion in patients with rectal cancer.

## Materials and Methods

### Patients

**Figure 1:**
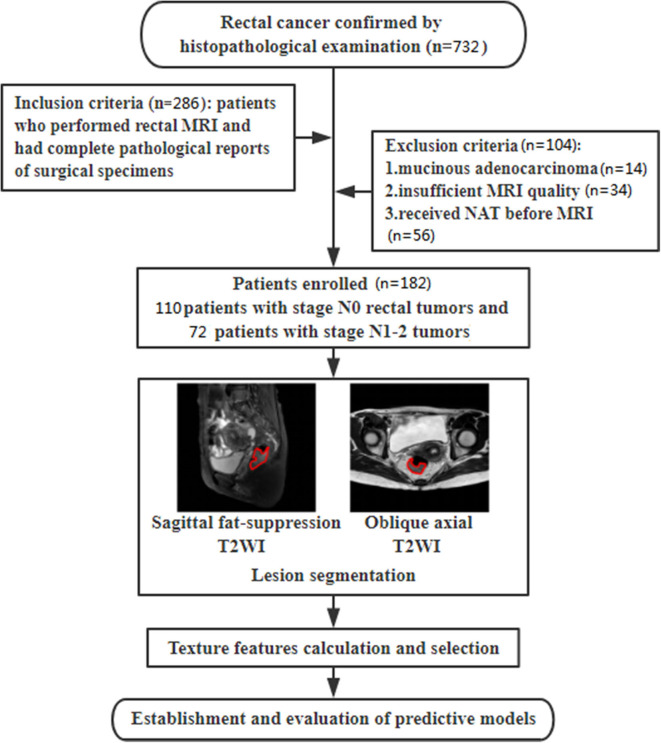
Flowchart of this study for discriminating N stage.

This retrospective study was approved by the ethics review board of Shengjing Hospital of China Medical University (2020PS011K) and the requirement for informed consent was waived. Between September 2018 and December 2019, records for 732 consecutive patients with histopathologically confirmed rectal cancer were initially retrieved using the picture archiving and communication system (PACS) in our institution. A series of 286 patients were enrolled in this study. Inclusion criteria were as follows: (1) patients who underwent high-resolution preoperative rectal MRI examinations; (2) histopathologically confirmed rectal cancer; (3) a time interval between MRI scanning and radical resection of <1 month. Clinical characteristics including gender, age, tumor location, maximum diameter of tumor, degree of tumor differentiation, and T stage were also collected using PACS. One hundred and four patients were excluded for the following reasons: (1) pathologically proven mucinous adenocarcinoma (*n* = 14); (2) insufficient MRI quality due to obvious motion artifacts caused by respiration or intestinal peristalsis (*n* = 34); and (3) received NAT before MRI scanning (*n* = 56). Finally, 182 patients with a median age at diagnosis of 61 years (age range, 54–92 years), including 120 (65.9%) men and 62 (34.1%) women, were collected and analyzed in this retrospective study. Patients were divided into low N stage (stage N0) and high N stage (stage N1-2) groups according to the results of histopathological examinations. The flowchart of this study is shown in Figure 1.

### MRI Scanning Protocol

All patients underwent preoperative rectal MRI in the supine position using a 3.0-T scanner (Ingenia 3.0, Philips Medical System, Best, The Netherlands) using an eight-channel phased-array surface coil. No bowel preparation or intravenous antispasmodics were used before the examination. The high-resolution rectal MRI protocol consisted of sagittal T2WI, oblique axial T2WI, oblique coronal T2WI, sagittal fat-suppression T2WI sequences and oblique axial diffusion-weighted imaging (DWI) with two b-factors (0 and 1,000 s/mm^2^). Oblique axial and oblique coronal T2WI were acquired perpendicular and parallel to the long axis of the tumor as identified on sagittal images. Acquisition parameters were: repetition time/echo time, 6,000/76 ms; flip angle, 90°; matrix size, 288 × 288; field of view, 450 mm; slices, 48; slice thickness, 5 mm; spacing between slices, 1 mm. All MRI images were retrieved from PACS for further analysis.

### Lesion Segmentation

For each patient, two radiologists with more than 8 years of experience in interpreting pelvic MRI who were blinded to pathological results independently determined the region of interest (ROI) of rectal tumors. Rectal cancers were determined as local masses or abnormal wall thickening that showed intermediate intensity of signal on T2WI, hyperintensity on DWI and hypointensity on apparent diffusion coefficient (ADC) maps. Contours of rectal tumor ROIs were manually delineated along the lesion area border on a single slice of sagittal fat-suppression and axial T2WI that showed the maximum tumor diameter with reference to the corresponding DWI and ADC maps using image processing software (ImageJ, National Institutes of Health, MD). To minimize bias, obvious necrosis, gas and lumen content areas were excluded.

### Texture Analysis

The sagittal fat-suppression and axial T2WI with manually delineated ROIs were imported into the MATLAB 2018a (Mathworks, Natick, MA, USA). All pixel intensities within the ROI were normalized between μ ± 3σ (μ: mean of image intensity within the ROI; σ: standard deviation), and the range was quantized to 8 bits/pixel. Ten texture features were extracted automatically from ROIs on sagittal fat-suppression and oblique axial T2WI images using an in-house software programmed with MATLAB. Texture features were divided into three feature groups: (1) histogram; (2) gray-level co-occurrence matrix (GLCM); and (3) gray-level run-length matrix (GRLM). A total of 20 texture features were calculated for each patient. Detailed information about extracted features is in Table 1.

**Table 1:** Definitions of extracted features.

**Feature**	**Definition**
**Histogram**	
Skewness (SKE)	Asymmetry of intensity level distribution
Kurtosis (KUR)	Peakedness of intensity level distribution
**GLCM parameters**^**a**^	
Contrast (CON)	Local variations presented in an image
Energy (ENE)	Uniformity of the image gray level distribution
Entropy (ENT)	Randomness of the intensity distribution
Information correlation (INC)	Non-linear gray level dependence
**GRLM parameters**^**b**^	
Long run emphasis (LRE)	Measures distribution of long runs
Run length non-uniformity (RLN)	Measures the similarity of the length of runs through out the image
Low gray-level run emphasis (LGLRE)	Measures the distribution of low gray level values
Short run low gray-level emphasis (SRLGLE)	Measures the joint distribution of short runs and low gray level values

a *GLCM, gray level co-occurrence matrix; Texture features were calculated from four GLCMs (corresponding to a distance of one pixel, and four angles 0, 45, 90, and 135°). Mean value of each feature over the four GLCMs was utilized*.

b *GRLM, gray level run-length matrix; Texture features were calculated from four GRLMs (corresponding to four angle 0, 45, 90, and 135°). Mean value of each feature over the four GRLMs was utilized*.

### Statistical Analysis

For categorical variables of gender, tumor location, degree of tumor differentiation, and T stage, chi-square or Fisher's exact test was performed between N0 and N1-2 groups. For quantitative data of age, maximum diameter of tumor, and texture parameters, independent sample *t*-test or Mann-Whitney *U*-test was performed between two groups after using the Kolmogorov-Smirnov test for normality analysis. Multivariate logistic regression analysis using forward stepwise selection was applied with entry of variables to identify independent factors for N1-2 tumors. Diagnostic performances of parameters for predicting stage N1-2 tumors were assessed using receiver operating characteristic (ROC) curve analysis, in which the area under the ROC curve (AUC), sensitivity, and specificity were measured. The optimal threshold was chosen according to the Youden index. Interobserver agreement for measurements of the two radiologists was determined by calculating intraclass correlation coefficients (ICCs, 0–0.4, poor agreement; 0.41–0.6, moderate agreement; 0.61–0.8, good agreement; 0.81–1, excellent agreement). Spearman correlation analysis was performed to evaluate correlations between texture features and N stages. *P* < 0.05 were considered statistically significant. All statistical analyses were performed using SPSS 22.0 (IBM, Corp) and MedCalc (version 14.10.20, http://www.medcalc.org).

## Results

### Patient Profiles

**Figure 2:**
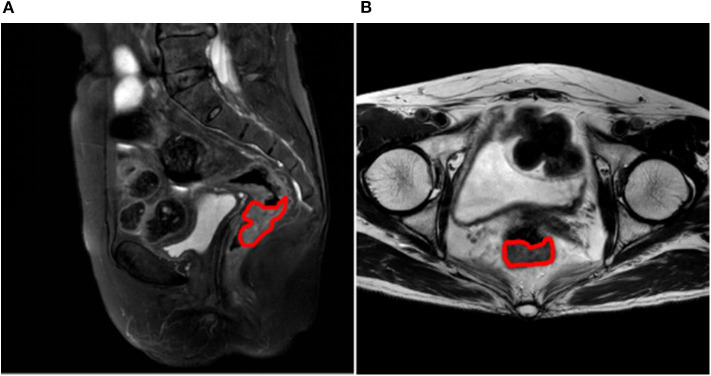
**(A)** Lesion segmentation result on sagittal fat-suppression T2WI, the margin of the lesion is marked in red; **(B)** Lesion segmentation result on oblique axial T2WI, the margin of the lesion is marked in red.

Patient profiles, including clinical and pathological characteristics for stage N0 and N1-2 patients are shown in Table 2. No significant differences between the two groups were seen for characteristics of gender (*P* = 0.905), age (*P* = 0.614), tumor location (*P* = 0.284), maximum diameter of tumor (*P* = 0.317), degree of tumor differentiation (*P* = 0.138), or T stage (*P* = 0.116). A randomly selected case illustrates the results of lesion segmentation in Figure 2.

**Table 2:** Clinical and pathological characteristics of patients.

**Characteristic**	**All**	**N stage**		***P*-value**
		**N0**	**N1-2**	
Total patients	182	110 (60.4)	72 (39.6)	/
Gender				0.905^a^
Male	120	72 (39.5)	48 (26.4)	
Female	62	38 (20.9)	24 (13.2)	
Mean age (years)	62.2 (54-92)	61.8 (54-83)	62.5 (57-92)	0.614^b^
Tumor location				0.284^c^
Upper rectum	54	26 (14.3)	28 (15.4)	
Middle rectum	102	66 (36.3)	36 (19.8)	
Lower rectum	26	18 (9.9)	8 (4.3)	
Maximum diameter of tumor (cm)	3.67	3.64	3.72	0.317^d^
Tumor differentiation				0.138^a^
Moderate to high	152	98 (53.8)	54 (29.7)	
Low	30	12 (6.6)	18 (9.9)	
T stage				0.116^a^
T1-2	144	94 (51.6)	50 (27.5)	
T3-4	38	16 (8.8)	22 (12.1)	

a *Variables were tested using the χ^2^ test*.

b *Variables were tested using independent sample t-test, data are mean value (range)*.

c *Variables were tested using Fisher's exact test*.

d *Variables were tested using Mann-Whitney U-test, data are median*.

*Unless otherwise indicated, variables are expressed as frequencies (percentage)*.

### Interobserver Agreement

Interobserver agreement between the two radiologists was excellent for all texture features derived from separately delineated ROIs (ICCs ranged from 0.852 to 0.934). Therefore, only results of the first radiologist were used for subsequent analysis.

### Univariate Analysis

**Figure 3:**
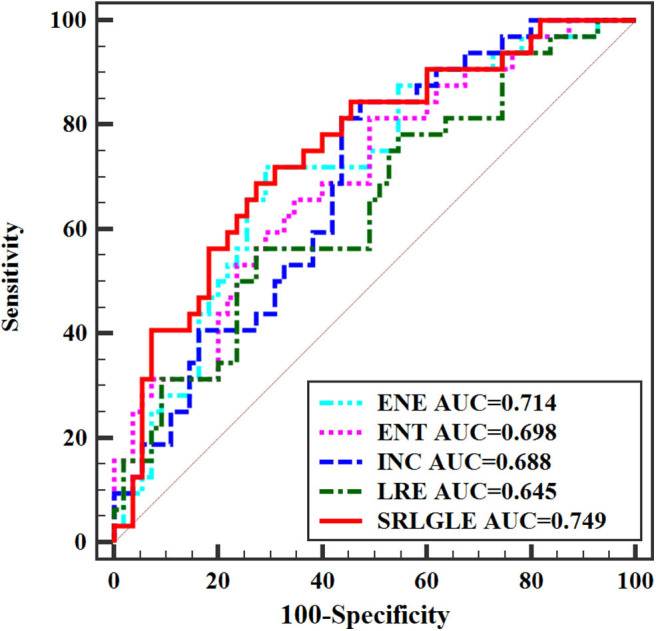
ROC curves of statistically significant texture features extracted from sagittal fat-suppression T2WI for predicting N stage.

**Figure 4:**
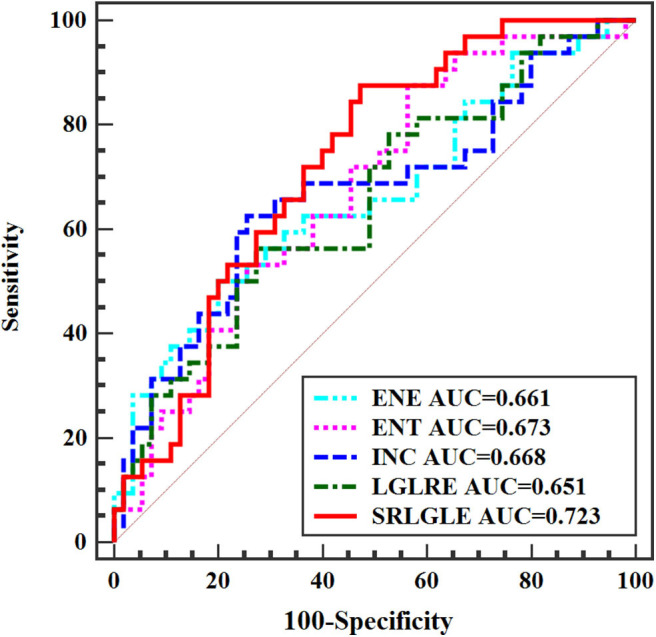
ROC curves of statistically significant texture features extracted from oblique axial T2WI for predicting N stage.

Differences in extracted texture features between stage N0 and N1-2 tumors are in Table 3. For features derived from sagittal fat-suppression T2WI images, a significantly lower ENE was found for stage N1-2 compared to stage N0 tumors. ENT, INC, LRE, and SRLGLE were significantly higher in the stage N1-2 than the N0 group. For features extracted from oblique axial T2WI images, stage N1-2 tumors had a significantly lower ENE and LGLRE and higher ENT, INC, and SRLGLE than stage N0 tumors. Diagnostic performance of significant texture features for discriminating N stage of rectal tumors are shown in Table 4. Corresponding ROC curves are in Figures 3, 4.

**Table 3:** Differences in texture features between stage N0 and N1-2 groups.

**MRI images**	**Feature**	**N stage**		***P*-value**
		**N0**	**N1-2**	
Sagittal fat-suppression T2WI	SKE	0.112 ± 0.356	0.104 ± 0.331	0.162^a^
	KUR	3.054 ± 0.578	3.246 ± 0.718	0.191^a^
	CON	0.399 ± 0.166	0.457 ± 0.224	0.198^a^
	ENE	0.973 ± 0.023	0.035 ± 0.017	**0.001**^**b**^
	ENT	0.135 ± 0.107	0.958 ± 0.032	**0.002**^**b**^
	INC	0.202 ± 0.142	0.377 ± 0.115	**0.004**^**b**^
	LRE	2.394 ± 0.497	2.701 ± 0.659	**0.031**^**a**^
	RLN	3.372 ± 0.462	3.566 ± 0.549	0.068^b^
	LGLRE	0.546 ± 0.082	0.576 ± 0.072	0.079^a^
	SRLGLE	0.035 ± 0.009	0.044 ± 0.013	**0.001**^**a**^
Oblique axial T2WI	SKE	0.479 ± 0.686	0.596 ± 0.529	0.374^a^
	KUR	5.129 ± 2.783	5.172 ± 2.093	0.936^a^
	CON	0.304 ± 0.112	0.348 ± 0.136	0.119^a^
	ENE	0.979 ± 0.011	0.975 ± 0.019	**0.013**^**b**^
	ENT	0.106 ± 0.049	0.132 ± 0.086	**0.007**^**b**^
	INC	0.321 ± 0.076	0.348 ± 0.108	**0.009**^**b**^
	LRE	1.722 ± 0.544	1.609 ± 0.498	0.327^a^
	RLN	3.187 ± 0.205	3.171 ± 0.324	0.902^b^
	LGLRE	0.578 ± 0.054	0.549 ± 0.068	**0.014**^**a**^
	SRLGLE	0.059 ± 0.024	0.062 ± 0.021	**0.001**^**a**^

a *Independent sample t-test. Data are mean ± standard deviation*.

b *Mann-Whitney U-test. Data are median ± interquartile range*.

**Table 4:** Diagnostic performance of statistically significant texture features for differentiating stage N0 and N1-2 tumors.

**MRI images**	**Feature**	**AUC^**a**^**	**Sensitivity (%)**	**Specificity (%)**	**95% CI^**b**^**	**Cutoff value**
Sagittal fat-suppression T2WI	ENE	0.714	71.87	70.91	0.607–0.806	< 0.944
	ENT	0.698	81.25	50.91	0.591–0.792	> 0.215
	INC	0.688	81.25	56.36	0.579–0.783	> 0.473
	LRE	0.645	56.25	72.73	0.535–0.745	> 2.867
	SRLGLE	0.749	68.75	72.73	0.645–0.836	> 0.004
Oblique axial T2WI	ENE	0.661	50.01	80.02	0.551–0.759	< 0.978
	ENT	0.673	80.52	43.64	0.564–0.770	> 0.107
	INC	0.668	62.50	74.55	0.558–0.765	> 0.321
	LGLRE	0.651	56.25	72.73	0.541–0.750	< 0.569
	SRLGLE	0.723	87.51	52.73	0.616–0.813	> 0.006

a *AUC, area under the receiver operating characteristic curve*.

b *CI, confidence interval*.

### Multivariate Analysis

Multivariate logistic regression analysis results and Spearman correlation coefficients are shown in Table 5. Five significant texture features extracted from sagittal fat-suppression T2WI of ENE, ENT, INC, LRE, and SRLGLE were entered in a logistic regression model using forward stepwise selection. Lower ENE and higher SRLGLE were independent predictors of lymph node invasion (stage N1-2). A logistic regression model used to differentiate stage N1-2 tumors from stage N0 tumors showed good accuracy of 78.56% with AUC 0.759, sensitivity 78.12%, specificity 74.55%, and goodness of fit (*P*-value of 0.131) by the Hosmer and Lemeshow test. Five significant texture features derived from oblique axial T2WI (ENE, ENT, INC, LGLRE, and SRLGLE), were entered in a logistic model using forward stepwise selection. Logistic regression analysis demonstrated that higher INC and SRLGLE were independent predictors. The model showed moderate accuracy of 72.48% with AUC 0.747, sensitivity 68.75%, specificity 78.18%, and goodness of fit (*P*-value of 0.351) in the Hosmer and Lemeshow test. In addition, SRLGLE derived from sagittal fat-suppression T2WI showed the strongest correlation with N stage (*r*_s_ = 0.417) among all independent predictors.

**Table 5:** Multivariate logical regression analysis results.

**MRI images**	**Feature**	**OR^**a**^**	**95% CI^**b**^**	***P*-value**	**Correlation with N stage (*r*_**s**_)**
Sagittal fat-suppression T2WI	ENE	3.93	1.146–9.991	0.027	0.357
	SRLGLE	3.02	1.028–8.851	0.044	0.417
Oblique axial T2WI	INC	4.274	1.575–11.494	0.004	0.280
	SRLGLE	5.882	1.742–19.231	0.004	0.372

a *OR, odds ratio*.

b *CI, confidence interval*.

**Figure 5:**
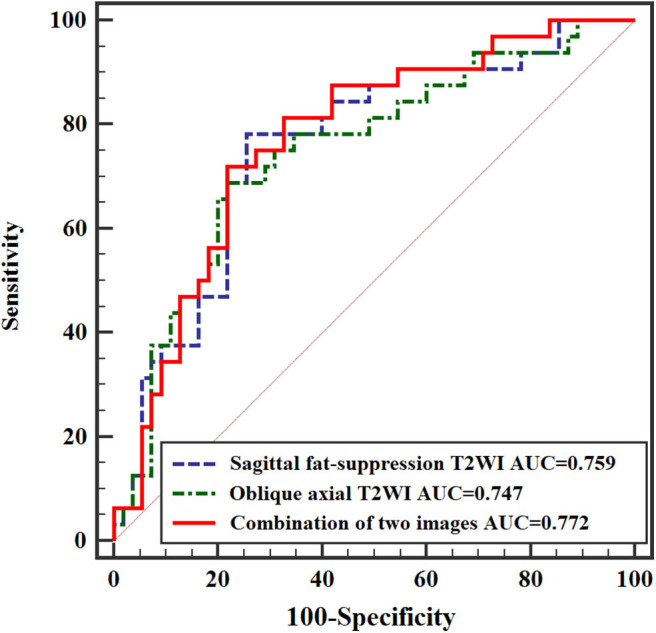
ROC curves of independent predictors for predicting N stage.

When significant texture features extracted from sagittal fat-suppression T2WI were combined with significant features extracted from oblique axial T2WI and used in the logistic regression model, lower ENE from sagittal fat-suppression T2WI and higher INC and SRLGLE from oblique axial T2WI were found to be independent predictors. The model showed good accuracy of 74.41% with AUC 0.772, sensitivity 71.87%, specificity 78.29%, and goodness of fit (*P*-value of 0.402) in the Hosmer and Lemeshow test. ROC curves are shown in Figure 5.

## Discussion

This study performed a texture analysis of rectal tumors on preoperative sagittal fat-suppression T2WI and oblique axial T2WI, and investigated correlations between texture features and lymph node invasion status. The results revealed that texture features derived from T2WI were valuable in predicting N stage for patients with rectal cancer.

Lymph node invasion status may be an indicator for NAT before surgery in patients with rectal cancer. Compared with surgery alone, use of NAT followed by surgical resection for locally advanced rectal carcinoma (stage N1-2 and/or T3-4) is associated with a 50-61% reduction in the risk of local recurrence (24, 25). Nonetheless, preoperative detection of lymph node invasion remains a challenge (6, 26). Previous studies used size as a criterion for assessing lymph node invasion, but size cutoff values used to distinguish between benign and malignant lymph nodes were not consistent, and metastases may occur with normal-sized lymph nodes (27). In our study, histogram and second-order texture features of rectal tumor were used to predict lymph node invasion status. Texture analysis allows quantification of intratumoral heterogeneity caused by variations in cellularity, angiogenesis, and extravascular extracellular matrix, as well as areas of hemorrhage and necrosis within tumors (28). Jalil et al. (29) demonstrated that texture features extracted from T2WI of rectal cancers were indicators of tumor response to NAT and long-term survival. A study by Liu et al. (30) showed that histogram texture features derived from ADC maps were associated with extramural invasion in rectal tumors. Yang et al. (23) found that patients in a negative regional lymph node metastasis group had significant higher skewness, kurtosis and energy, and lower entropy of tumors on T2WI than in a positive group. However, the study by Yang et al. (23) contained only histogram texture features. Therefore, the correlation between second-order texture features and lymph nodes invasion status is still unclear, and no previous studies were found to report that unknown scientific problem.

In this study, a significantly lower ENE and higher ENT, INC, LRE, and SRLGLE extracted from sagittal fat-suppression T2WI images were found for stage N1-2 compared to stage N0 rectal tumors. Stage N1-2 tumors had significantly lower ENE and LGLRE, and higher ENT, INC, and SRLGLE derived from oblique axial T2WI images than stage N0 tumors. ROC curves showed that SRLGLE extracted from sagittal fat-suppression T2WI had the best performance for predicting lymph node invasion among all significant features with the cutoff value of 0.004, yielding an AUC of 0.749, moderate sensitivity of 68.75% and moderate specificity of 72.73%. Higher ENE reflects a more uniform distribution of gray levels. ENT is the opposite index to ENE. Higher LRE and SRLGLE reflect greater disorder of the distribution of long runs and the joint distribution of short runs and low gray-level values. As a result, the variation in these texture features in our study showed that rectal tumors with high N stage might have more heterogeneity and higher texture complexity on T2WI than tumors with lower N stage.

Multivariate logistic regression analysis further demonstrated the association between texture features and rectal cancer N stage. A model with significant texture features extracted from sagittal fat-suppression T2WI combined with significant features extracted from oblique axial T2WI showed that lower ENE from sagittal fat-suppression T2WI and higher INC and SRLGLE from oblique axial T2WI were independent predictors. Yang et al. (23) performed texture analysis on axial T2WI and found that ENE was different for stage N0 and N1-2 rectal tumors, but ENE was not an independent predictor of N stage. This result may be because different institutions use different MRI technologies and parameters, resulting in variation in texture features for T2WI images. In addition, the AUC from the logistic regression model combining texture features extracted from two kinds of MRI images was higher than the AUCs from models of texture features derived from sagittal fat-suppression T2WI or oblique axial T2WI for discriminating lymph node invasion status in rectal cancer.

Interobserver agreement for texture features extracted from both sagittal fat-suppression T2WI and oblique axial T2WI was also evaluated. The results indicated excellent agreement between the two radiologists for calculating texture features based on a single-slice method. ICCs ranged from 0.852 to 0.934. Interobserver variability mainly originated from slice image selection and ROI delineation because our next step was calculating texture features within the ROI using in-house software in MATLAB. Thus, standard strategies for ROI definition and delineation are important.

The study had several limitations. First, the lack of multi-institutional validation of texture features as well as the relatively small sample size may impede the generalizability of the findings. A large sample size and multi-institution cohort is necessary to further validate the results of our present study. Second, only a single slice with the maximum tumor diameter was applied to extract features in this study. Analysis based on one single slice would inevitably lose a lot of important information because of heterogeneity in tumor volume. Unlike solid organ tumors, rectal cancer usually grows along the rectal wall and forms an irregular shape. Texture analysis based on 3D volume images could more accurately represent the actual whole tumor volume and might be one of the strategies to improve the predictive.

Performance in identifying different lymph node invasion status (31). Third, only texture features were used for establishing predictive model to predict N stage of rectal tumors while clinical variables were not incorporated in the model. Some clinical information such as pretreatment carcinoembryonic antigen (CEA) data and pretreatment carbohydrate antigen 199 (CA199) data were revealed significantly different between metastasis and non-metastasis groups (32). In the future, clinical variables will be considered for incorporating into the model. Moreover, features were only extracted from sagittal fat-suppression and oblique axial T2WI. DWI and ADC maps were not included in this study. Liu et al. (30) revealed that texture features calculated from DWI or ADC maps were valuable for discriminating different T stage or N stage of rectal cancer. Further study can focus on DWI and ADC maps. Finally, the findings may not apply to advanced rectal cancer because we analyzed only patients who had surgical resection directly rather than those who first received NAT.

## Conclusions

In conclusion, texture features based on preoperative T2WI may be valuable for identifying lymph node invasion status in rectal cancer. In particular, lower ENE extracted from sagittal fat-suppression T2WI and higher INC and SRLGLE extracted from oblique axial T2WI were independent predictors of lymph node invasion in this study. Our results may provide useful information to help tailor treatment strategies.

## Data Availability Statement

Data cannot be shared publicly because data contain potentially identifying or sensitive patient information (name, birthday, ID, sex). Interested readers can request the data by contacting the corresponding authors (yinjd@sj-hospital.org). As the original dataset contains variables coded in China, interested readers may specifically request a recorded version of the data set when requesting the data as an aid to conducting analysis.

## Ethics Statement

The studies involving human participants were reviewed and approved by Shengjing Hospital of China Medical University. Written informed consent for participation was not required for this study in accordance with the national legislation and the institutional requirements.

## Author Contributions

LS analyzed the patient data and wrote the paper. JY was a major contributor in designing the manuscript. All authors read and approved the final manuscript.

## Conflict of Interest

The authors declare that the research was conducted in the absence of any commercial or financial relationships that could be construed as a potential conflict of interest.
